# Incidental detection of retinal nerve fiber layer thinning with slowed progression following optic nerve-carotid artery decompression: A case report

**DOI:** 10.1016/j.ajoc.2025.102376

**Published:** 2025-07-08

**Authors:** Hidenori Takahashi, Keisuke Ohtani, Takeshi Hara, Kensuke Kawai

**Affiliations:** a Center for Cyber Medicine Research, University of Tsukuba, 1-1-1 Tennodai Tsukuba-shi, Ibaraki, Japan; b Department of Ophthalmology, Jichi Medical University, 3311-1 Yakushiji Shimotsuke-shi, Tochigi, Japan; c Department of Neurosurgery, Jichi Medical University, 3311-1 Yakushiji Shimotsuke-shi, Tochigi, Japan; d Hara Eye Hospital, 1-1-11 Nishi Utsunomiya-shi, Tochigi, Japan

**Keywords:** Neurovascular compression, Optic nerve, Internal carotid artery, Retinal nerve fiber layer thinning, Vascular decompression surgery, Case report

## Abstract

**Purpose:**

This report aims to present a case of the retinal nerve fiber layer (NFL) thinning, in which the progression of thinning was slowed following optic nerve-carotid artery decompression surgery.

**Observations:**

A 41-year-old ophthalmologist incidentally noticed a generalized thinning of NFL in his left eye using optical coherence tomography (OCT). Five years earlier, the OCT showed only mild thinning. While visual acuity and visual fields were normal, the patient reported a blurriness. The MRI revealed an ectatic and tortuous left internal carotid artery compressing the left optic nerve. Due to the lack of reports on the correlation between NFL thickness and visual acuity, an internal review was conducted. We searched for cases with a progression from healthy visual acuity to blindness, and in the only case identified, we investigated the correlation between NFL thickness and visual acuity. Applying this correlation, it was predicted that the patient would lose vision by age 48. As a result, the patient decided to receive decompression surgery. Following the surgery, the rate of NFL thinning significantly slowed from −1.9 μm/year to −0.57 μm/year (p < .0001). At the current age of 48, the patient's corrected visual acuity is 20/16, and visual field remains normal. The predicted onset of blindness has been postponed by 10 years.

**Conclusions and importance:**

We report a case in which compression of the optic nerve by the internal carotid artery was observed in a patient with retinal nerve fiber layer (NFL) thinning. A slowing of NFL thinning was noted following surgical decompression.

## 1 Introduction

Neurovascular compression syndromes are caused by the compression of cranial nerves by adjacent intracranial blood vessels.^1^ This report presents a case in which the optic nerve (CN2) is compressed by the internal carotid artery, leading to progressive thinning of the retinal nerve fiber layer (NFL).

Common neurovascular compression syndromes include hemifacial spasm, trigeminal neuralgia, glossopharyngeal neuralgia, spasmodic torticollis, and oculomotor nerve palsy, among others. These conditions are generally associated with thinner cranial nerves. Reports of visual impairment caused by vascular compression of the thicker CN2, are rare.^2^ Notable cases include a report of visual field defect resolution following vascular decompression surgery^3^ and a study indicating a higher prevalence of internal carotid artery contact in normal-tension glaucoma.^4^

In most cases of trigeminal neuralgia, symptoms can be alleviated in approximately 90 % of patients through microvascular decompression surgery, which relieves the pressure on the affected nerve.^1^ In the present report, following vascular decompression surgery, the rate of NFL thinning slowed by 70 %.

## 2 Case report

**Fig. 1:**
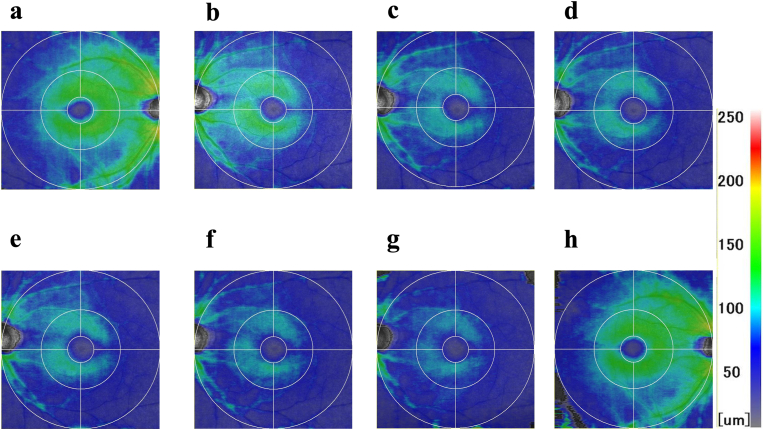
ILM–IPL/INL thickness maps obtained using RS-3000 OCT. A: Left eye at age 36. B–G: Right eye before and after surgery at ages 36, 40, 42, 42, 45, and 48, respectively. H: Left eye at age 48. The scale on the right represents the color-coded thickness. (For interpretation of the references to color in this figure legend, the reader is referred to the Web version of this article.)

A 41-year-old Japanese ophthalmologist, the first author of this manuscript, underwent optical coherence tomography (OCT) imaging using RS-3000 (Nidek Inc., Aichi, Japan) and noticed a generalized thinning of NFL in the left eye (Fig. 1C).

He had himself imaged using various devices to personally experience what patients undergo during their medical examinations. Five years earlier, when the first author of this manuscript was appointed to the hospital, imaging with the same OCT device revealed mild thinning of NFL (Fig. 1A and B). While best corrected visual acuity (BCVA) was 20/10, visual fields, the Panel D-15, and Ishihara tests were normal, left relative afferent pupillary defect was detected, the patient began to experience blurriness outside the central vision in the left eye, and this symptom progressively worsened over time. Compared to the right eye, the visual blurriness in the left eye increased.

His equivalent spherical power was −5.1D. The intraocular pressure was 14 mmHg in both eyes. He had no medical history other than myopia, atopic dermatitis, and childhood asthma, and no abnormalities had ever been noted in the anterior segment or media. In particular, there were no clinical signs suggestive of keratoconus. His BMI was 19.0, calculated from a height of 173 cm and weight of 57 kg. His average blood pressure was 113/72 mmHg, and his heart rate was 66 beats per minute. The patient was positive for HLA-DR4. His family history included VKH-like uveitis in his mother (who was HLA-DR4 negative), and high myopia in both parents, his brother, and his two sons.

Initially suspected to have primary open-angle glaucoma, the presentation was atypical, prompting an MRI to investigate intracranial causes. Based on the diagnosis of glaucoma, the patient was treated with prostaglandin analog eye drops, which temporarily resulted in a reduction in interpupillary distance. The intraocular pressure decreased to as low as 8 mmHg at its most effective point. The changes in interpupillary distance over time were previously published in a study.^5^

**Fig. 2:**
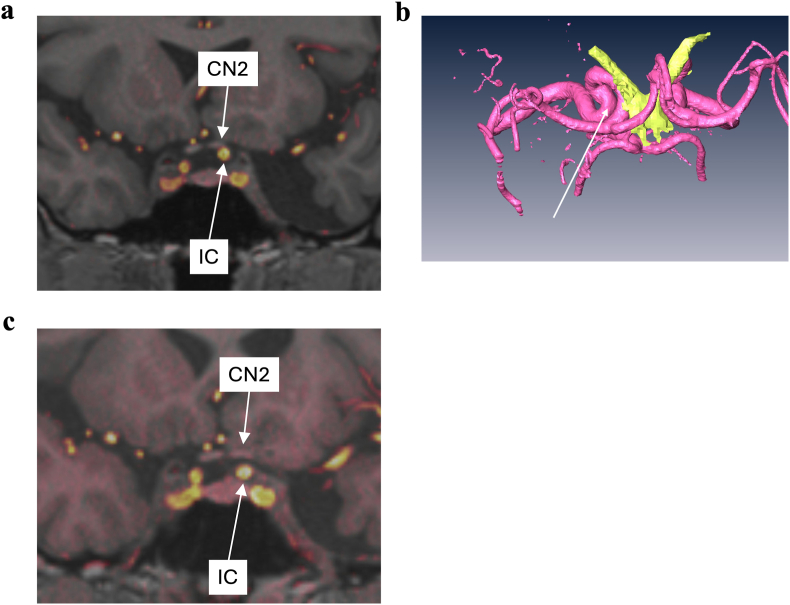
Preoperative and postoperative MRI–CT fusion images. A: Pre-operative MRI–CT fusion image showing compression of the left optic nerve (CN2) from bellow by the internal carotid artery (IC). B: 3D reconstruction confirmed The same finding. C: Postoperative MRI–CT fusion image demonstrating a clear space between the optic nerve and the internal carotid artery, indicating that the compression has been released.

The MRI revealed an ectatic and tortuous left internal carotid artery (C1-2 segment) compressing and elevating the left optic nerve and the left side of the optic chiasm caudally. The fusion image of MRI and CT showed that the optic nerve was being compressed from below by the internal carotid artery. There was no atrophy dorsally or laterally from the compression site to the lateral geniculate body, suggesting that vascular compression was the primary cause rather than secondary degeneration (Fig. 2A and B).

The first author of this manuscript wanted to estimate the age at which he would lose visual function in one eye and compare it with the age until which he hoped to continue cataract and vitreoretinal surgery. A PubMed search was conducted for a regression equation correlating NFL thickness with BCVA, but although several studies examined this correlation,^6^, ^7^, ^8^ no regression formula was found. Therefore, an internal review was conducted.

We searched for cases that progressed from a normal BCVA to blindness and identified only one such case: a patient with secondary glaucoma due to refractory uveitis who lost vision over five years. The spherical equivalent power of this patient was −18D.

**Fig. 3:**
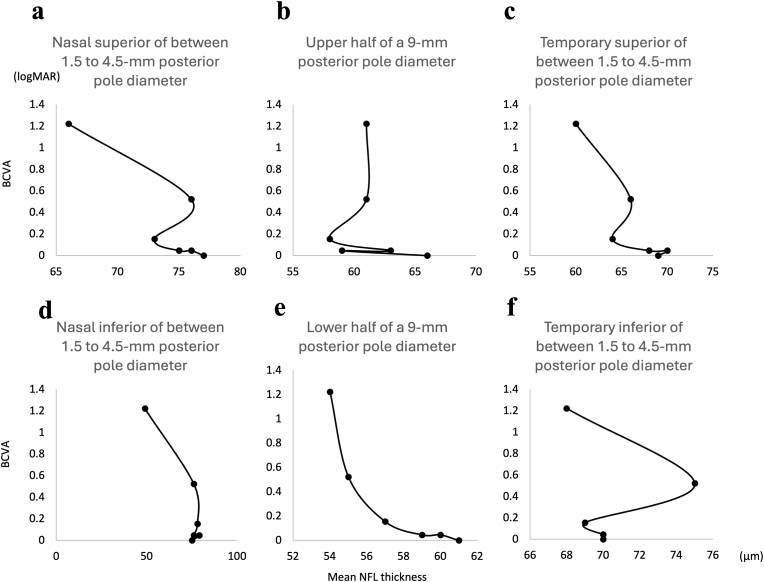
Correlations between NFL thicknesses and BCVA in a patient with secondary glaucoma due to refractory uveitis, who had lost vision over five years. The lower half of a 9-mm posterior pole diameter (E) revealed the clearest correlation.

A study of NFL thicknesses across various retinal area and visual acuity in this patient revealed a clear correlation between mean NFL thickness in the lower half of a 9-mm posterior pole diameter and visual acuity (Fig. 3). The conversion formula was “logMAR BCVA = 1/(mean NFL thickness in the lower half of a 9-mm posterior pole diameter (μm) - 53.252388854) - 0.1061256”. This means that when the mean NFL thickness in the lower half of a 9-mm posterior pole diameter approaches 53 μm, the predicted logMAR BCVA diverges, suggesting a complete visual loss.

**Fig. 4:**
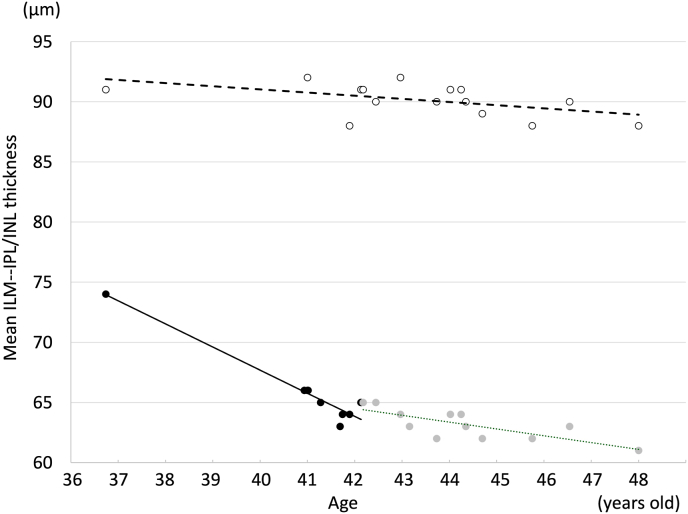
Course of mean NFL thickness of a lower half of a 9-mm posterior pole diameter. White dots: Left eye. Black dots: Right eye before surgery. Gray dots: Right eye after surgery. Following the surgery, the rate of NFL thinning of left eye significantly slowed from −1.9 μm/year to −0.57 μm/year (p < .0001). The rate of right is −0.26 μm/year.

Assuming a linear decline in NFL thickness, and applying this correlation, it was predicted that the patient would lose vision by age 48 (Fig. 4, solid line). As a result, the first author requested neurosurgeons to perform decompression of the internal carotid artery. At that time, neither the neurosurgeons suggested nor the first author considered the use of anticoagulants or antihypertensive agents to relieve the compression.

### 2.1 Intraoperative report

Recently, endoscopic endonasal approaches have become increasingly popular for optic canal decompression. However, in this case, due to the anatomical relationship between the internal carotid artery (ICA) and the optic nerve (ON), an endonasal approach was deemed inappropriate. First, in the event of ICA injury, adequate hemostasis and vascular control may be extremely difficult with an endonasal approach. Second, the ICA was compressing the optic nerve from the inferolateral side, which required decompression of the superior wall of the optic canal—a technically difficult maneuver through and endonasal approach. Moreover, transposition of the ICA using a graft material was attempted during surgery, which would not have been feasible through the sphenoid sinus alone. Therefore, a transcranial craniotomy was selected to ensure both adequate decompression and surgical safety.

Under general anesthesia, the patient was positioned supine, with the head rotated approximately 45° to the right and fixed at three points with the vertex lowered. A Navigation VEP, MEE-2000 (Nihon Kohden Co., Japan) setup was applied; however, stable waveforms could not be obtained from left eye stimulation (right eye stimulation showed good responses). An arcuate skin incision was made in the frontotemporal region, and the muscle-fascia flap was elevated and retracted anteriorly.

A frontotemporal craniotomy was performed through three burr holes. The sphenoid ridge was maximally removed. Dural tenting sutures were placed, and the dura was incised in an arcuate manner and reflected anteriorly.

A surgical microscope was introduced, and the Sylvian fissure was widely dissected from its distal portion, naturally exposing the basal cisterns. The origins of M1, IC, and A1 were identified and completely dissected. From a slightly lateral perspective, the IC portion C1-2 was observed to be compressing the optic nerve from an inferolateral to superior direction. The arachnoid between the optic nerve and IC was dissected. However, this alone did not allow sufficient mobilization of the IC, so as planned, the anterior clinoid process and proximal optic canal were opened.

The superior wall of the optic canal and the anterior clinoid process were removed. After cutting the IC distal dural ring, the mobility of both the IC and optic nerve improved significantly. Applying moderate pressure on the IC allowed the creation of space between it and the optic nerve, but decompression remained insufficient. Additionally, a hematoma-like aneurysm was noted immediately distal to the dural ring. Therefore, the IC was secured along with the arterial segment using a 5mm-wide PTFE(polytetrafluoroethylene)material, which was fixed at both ends with fibrin glue to the diaphragma sellae and free edge of the tentorium. This maneuver achieved significant decompression compared to the initial state, but the distance between the IC and optic nerve was not maximized when directly pressing the IC downward. Further manipulation risked IC kinking, and additional insertion of raised concerns about optic nerve compression, leading to termination of the procedure at this point.

The dural incision was sutured, and a fat graft harvested from the thigh (coated with fibrin glue) was inserted at the anterior clinoid process site to prevent cerebrospinal fluid leakage. The cranial bone flap was fixed with two round titanium plates and two bridging titanium plates. The fascia and subcutaneous layers were sutured, and the skin was closed with staples, concluding the surgery.

### 2.2 Postoperative course

Immediately after the surgery, diplopia due to abducens nerve palsy was observed, but it resolved in primary gaze within one week. Currently, diplopia occasionally occurs when looking to the left, but it is not bothersome in daily life. What is more noticeable is the scar on the thigh from the tendon extraction.

**Fig. 5:**
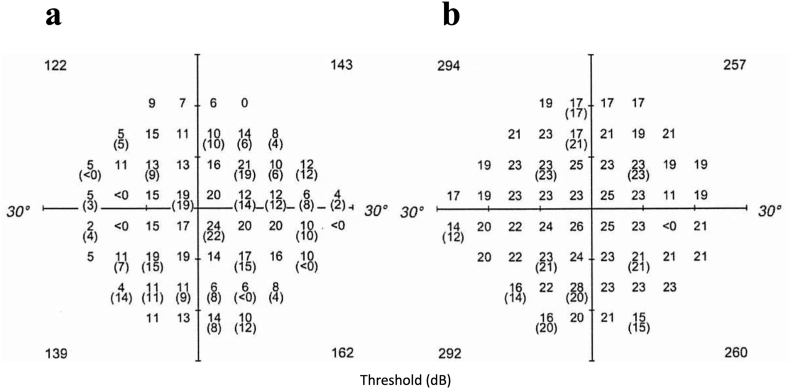
Humphrey Field Analyzer full threshold 24-2 using stimulus I. A: Left eye. B: Right eye. Foveal thresholds were 31 dB and 35 dB, respectively. The threshold of individual test points in the left eye were approximately 7dB lower than those in the right eye.

Following the surgery, the rate of NFL thinning significantly slowed from −1.9 μm/year to −0.57 μm/year (p < .0001) (Fig. 4). The rate of NFL thinning in the right eye is −0.26 μm/year (Fig. 4, dashed line). At the current age of 48, the patient's BCVA is 20/12. On Humphrey SITA Standard 10-2 testing, mean deviation (MD) was 0.35 dB in the left eye compared to 2.87 dB in the right eye, indicating a generalized reduction in sensitivity. To further evaluate subtle changes, full-threshold 24-2 testing was performed using size I stimulus, which revealed a more evident global decrease in sensitivity in the left eye (Fig. 5). Color vision was normal on both the Ishihara and Panel D-15 test, and the patient reported minimal subjective color difference between the two eyes.

Using the postoperative rate of NFL thinning and assuming a continued linear decline, the patient was estimated to lose vision at age 58 (Fig. 4, dotted line). This suggests that the surgery may have postponed the onset of complete visual loss by approximately 10 years.

From the author's perspective, this potential delay represents a meaningful outcome, even if the eventual loss of vision may be unavoidable.

## 3 Discussion

This case reports NFL thinning caused by optic nerve compression by the internal carotid artery and its long-term prognosis before and after decompression surgery, a rare occurrence.^1^, ^2^, ^3^ After conducting a literature review on February 9, 2025, utilizing PubMed using the key words ('optic neuropathy' and 'internal carotid artery'), as well as Google Scholar and chatGPT-o1 with the key words ('optic neuropathy', 'internal carotid artery', 'retinal nerve fiber layer', 'compression', '-traumatic', and '-retinal artery occlusion'), we did not find any prior reports of long-term (over 10 years) follow-up of optic neuropathy due to internal carotid artery compression specifically involving the retinal nerve fiber layer. In this case, detailed data could be obtained because the patient was the first author, who is an ophthalmologist.

In this case, the significant deceleration of NFL thinning following decompression surgery, when considered alongside the MRI findings, strongly suggests that the cause was indeed compression of the optic nerve by the internal carotid artery, as has been occasionally reported in the literature.^1^, ^2^, ^3^ Although thinning of the NFL continued even after decompression, the rate was approximately twice that of the fellow eye. This may be attributed to age-related thinning,^9^ accelerated degeneration due to prior axonal damage, or residual compression not evident on MRI, despite no re-contact being observed between the internal carotid artery and the optic nerve (Fig. 2C).

Although a significant correlation between NFL thickness and BCVA has been previously reported,^8^ the strength of this correlation is generally moderate and not necessarily linear. In the present internal review, we identified a single case in which the mean NFL thickness in the lower half of a 9-mm posterior pole diameter showed a remarkably strong correlation with visual acuity. However, this finding may have been coincidental.

Anatomically, retinal nerve fibers that project directly from the optic disc to the fovea are expected to be located in the upper half of the posterior pole. Therefore, if more cases were available, the upper half might have demonstrated a stronger structure-function relationship. On the other hand, developmental vulnerabilities such as thinner choroid in the inferior retina^10^—where the embryonic optic fissure closes—could support the observed lower-half correlation even in larger cohorts.

Furthermore, the thickness measurements relied on automatic segmentation, and the values are likely to differ if devices other than the Nidek RS-3000 are used.^11^ Therefore, the conversion formula between thickness and visual acuity lacks generalizability. In fact, measurements using the RS-3000 showed a thickness of 62 μm, while the same case measured 26 μm with the TOPCON Triton.

The RS-3000 device used in this study, despite being 15 years old since its manufacture, was still capable of capturing high-quality images. Since the measurements were performed on the same individual, the longitudinal data can be considered reliable.

The subjective symptoms of nerve compression are extremely subtle, and the surgeon advised against the operation, questioning its necessity given that both visual acuity and visual field were normal. However, as the first author regularly performs internal limiting membrane (ILM) peeling — a procedure that requires excellent binocular vision — the prospect of losing vision in one eye, even in the non-dominant eye, was professionally and personally unacceptable at the age of 40.

This study has several limitations.

First, as this is a single case report, even if similar NFL thinning is observed in other patients, it cannot be concluded that the cause is compression of the optic nerve by the internal carotid artery. Nor can decompression surgery be broadly recommended based on this case alone.

Second, the regression formula used to estimate visual acuity from NFL thickness is based on just one reference case and therefore lacks reliability. Moreover, the reference case in question likely experienced vision loss due to elevated intraocular pressure via a glaucomatous mechanism, rather than retrobulbar compression. As such, it may not be an appropriate benchmark for establishing general thresholds of blindness.

Additionally, axial length has been reported to influence NFL thickness, with longer eyes tending to show thinner NFL measurements.^9^^,^^12^ A correction for axial length may therefore be necessary.

## 4 Conclusions

This case suggests that compression of the optic nerve by the internal carotid artery may contribute to NFL thinning, and that surgical decompression may be associated with a reduction in the rate of thinning. The observed course in this patient supports a potential link between optic nerve compression and progressive 10.13039/100001267NFL loss, although further cases are needed to confirm this association.

## CRediT authorship contribution statement

**Hidenori Takahashi:** Writing – review & editing, Writing – original draft, Visualization, Software, Resources, Project administration, Methodology, Investigation, Formal analysis, Data curation, Conceptualization. **Keisuke Ohtani:** Writing – review & editing, Visualization, Software, Resources, Methodology, Investigation, Data curation. **Takeshi Hara:** Writing – review & editing, Validation, Supervision, Resources, Methodology, Investigation, Formal analysis, Data curation. **Kensuke Kawai:** Writing – review & editing, Validation, Supervision, Resources, Methodology.

## Patient consent

The data used in this case report were obtained during routine clinical practice, and the use of the data was approved by the institutional review board with an opt-out provision (approval number CU24-130).

## Acknowledgements and disclosures

No funding or grant support

## Authorship

All authors attest that they meet the current ICMJE criteria for Authorship.

## Declaration of generative AI and AI-assisted technologies in the writing process

Statement: During the preparation of this work the first author used chatGPT o1 to improve English language in this manuscript. After using this tool/service, the authors reviewed and edited the content as needed and take full responsibility for the content of the published article.

## Declaration of competing interest

The authors declare the following financial interests/personal relationships which may be considered as potential competing interests:Hidenori Takahashi reports a relationship with DeepEyeVision that includes: board membership, equity or stocks, and non-financial support. Hidenori Takahashi reports a relationship with 10.13039/501100004095Kyowa Kirin Inc that includes: funding grants, speaking and lecture fees, and travel reimbursement. Hidenori Takahashi reports a relationship with 10.13039/100008792Novartis Pharma KK that includes: funding grants, speaking and lecture fees, and travel reimbursement. Hidenori Takahashi reports a relationship with 10.13039/100015731Bayer Yakuhin Kabushiki Kaisha that includes: funding grants, speaking and lecture fees, and travel reimbursement. Hidenori Takahashi reports a relationship with Linical Corp that includes: funding grants. Hidenori Takahashi reports a relationship with Nippon Boehringer Ingelheim Co Ltd that includes: funding grants, speaking and lecture fees, and travel reimbursement. Hidenori Takahashi reports a relationship with 10.13039/501100004286Santen Pharmaceutical Co Ltd that includes: funding grants, speaking and lecture fees, and travel reimbursement. Hidenori Takahashi reports a relationship with Senju Pharmaceutical Co Ltd that includes: speaking and lecture fees and travel reimbursement. Hidenori Takahashi reports a relationship with 10.13039/100004334Merck & Co Inc that includes: speaking and lecture fees and travel reimbursement. Hidenori Takahashi reports a relationship with 10.13039/100010795Chugai Pharma Co Ltd that includes: speaking and lecture fees and travel reimbursement. Hidenori Takahashi reports a relationship with Hoya Corporation that includes: speaking and lecture fees. Hidenori Takahashi reports a relationship with Nikon Solutions Co Ltd that includes: speaking and lecture fees. Hidenori Takahashi has patent pending to DeepEyeVision. Hidenori Takahashi has patent issued to DeepEyeVision. Hidenori Takahashi has patent pending to Jichi Medical University. If there are other authors, they declare that they have no known competing financial interests or personal relationships that could have appeared to influence the work reported in this paper.
