# Supplementary material for: Incidental detection of retinal nerve fiber layer thinning with slowed progression following optic nerve-carotid artery decompression: A case report
Source: Am J Ophthalmol Case Rep. 2025 Jul 8;39:102376. doi: 10.1016/j.ajoc.2025.102376 (PMC12283895; doi:10.1016/j.ajoc.2025.102376)
Supplement: Multimedia component 1 [file mmc1.pdf]

CARE case report guidelines

CARE Checklist of information to include when writing a case report

(cc) BY-NC-ND

Topic	Item	Checklist item description	Reported on Line
Title	1	The diagnosis or intervention of primary focus followed by the words "case report"	1–2
Key Words	2	2 to 5 key words that identify diagnoses or interventions in this case report, including "case report"	45–46
Abstract (no references)	3a	Introduction: What is unique about this case and what does it add to the scientific literature?	24–26
	3b	Main symptoms and/or important clinical findings	27–32
	3c	The main diagnoses, therapeutic interventions, and outcomes	30–40
	3d	Conclusion—What is the main "take-away" lesson(s) from this case?	41–43
Introduction	4	One or two paragraphs summarizing why this case is unique (**may include references**)	58–59
Patient Information	5a	De-identified patient specific information.	NA
	5b	Primary concerns and symptoms of the patient.	68–70
	5c	Medical, family, and psycho-social history including relevant genetic information	70–79
	5d	Relevant past interventions with outcomes	89–92
Clinical Findings	6	Describe significant physical examination (PE) and important clinical findings.	80–89
Timeline	7	Historical and current information from this episode of care organized as a timeline	167–175
Diagnostic Assessment	8a	Diagnostic testing (such as PE, laboratory testing, imaging, surveys).	94–99
	8b	Diagnostic challenges (such as access to testing, financial, or cultural)	80–81
	8c	Diagnosis (including other diagnoses considered)	87–88
	8d	Prognosis (such as staging in oncology) where applicable	NA
Therapeutic Intervention	9a	Types of therapeutic intervention (such as pharmacologic, surgical, preventive, self-care)	119–121
	9b	Administration of therapeutic intervention (such as dosage, strength, duration)	123–164
	9c	Changes in therapeutic intervention (with rationale)	NA
Follow-up and Outcomes	10a	Clinician and patient-assessed outcomes (if available)	167–168
	10b	Important follow-up diagnostic and other test results	168–172
	10c	Intervention adherence and tolerability (How was this assessed?)	167–168
	10d	Adverse and unanticipated events	167-168
Discussion	11a	A scientific discussion of the strengths AND limitations associated with this case report	180–189
	11b	Discussion of the relevant medical literature **with references**.	191–198
	11c	The scientific rationale for any conclusions (including assessment of possible causes)	194–197
	11d	The primary "take-away" lessons of this case report (without references) in a one paragraph conclusion	260–270
Patient Perspective	12	The patient should share their perspective in one to two paragraphs on the treatment(s) they received	179-180
Informed Consent	13	Did the patient give informed consent? Please provide if requested	Yes ☑ No ☐
